# Regulation of ezrin tension by S-nitrosylation mediates non-small cell lung cancer invasion and metastasis: Erratum

**DOI:** 10.7150/thno.107681

**Published:** 2025-01-01

**Authors:** Xiaolong Zhang, Guangming Li, Yichen Guo, Ying Song, Linlin Chen, Qinli Ruan, Yifan Wang, Lixia Sun, Yunfeng Hu, Jingwen Zhou, Bin Ren, Jun Guo

**Affiliations:** 1 State Key Laboratory Cultivation Base for TCM Quality and Efficacy, School of Medicine and Life Science, Nanjing University of Chinese Medicine, Nanjing 210023, Jiangsu, PR China.; 2 Key Laboratory of Drug Target and Drug for Degenerative Disease, Nanjing University of Chinese Medicine, Nanjing 210023, Jiangsu, PR China.; 3 Department of Anesthesiology, Huaian First People's Hospital, Nanjing Medical University, Huaian 223001, Jiangsu, PR China.; 4 Department of Surgery and Biomedical Engineering, University of Alabama at Birmingham (UAB), Birmingham, Alabama. 35294, USA.; 5 Department of Respiratory Medicine, The First Affiliated Hospital of Nanjing Medical University, Nanjing 210029, PR China; 6 Affiliated Hospital of Nanjing University of Chinese Medicine, Nanjing 210023, Jiangsu, PR China.; 7 The First Clinical Medical College of Nanjing University of Chinese Medicine, Nanjing, Jiangsu 210023, PR China.

The authors would like to issue a correction regarding the figures in our published paper. We have identified an error that the Figure 4A Day 0-Ezrin-WT and Day 28-Ezrin-C117S might show the same mice. Accordingly, we revise the pictures in Figure 4A. This mistake occurred during data collection and final preparation of the figures.

We confirm that these corrections do not affect the results and main conclusions of the paper. The data in both the incorrect and correct figures are based on the same analysis, and there is no change to the results or interpretations presented in the study.

We apologize for any inconvenience caused by these errors and appreciate your understanding.

## Figures and Tables

**Figure 4:**
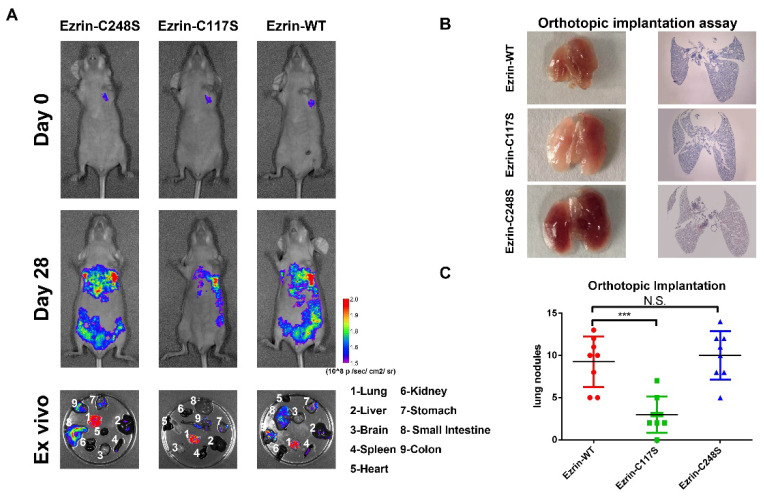
** Ezrin Cys^117^ correlates positively with NSCLC invasion and metastasis in vivo. (A)** For the orthotopic implantation assay, bioluminescent images of systemic metastases in nude mice with ezrin-WT, ezrin-C117S, and ezrin-C248S A549 cells are shown. The ex vivo organ metastases including those in the lungs, liver, brain, spleen, heart, kidney, stomach, small intestine, and colon were also presented.** (B)** Representative images of H&E-stained histological sections of lungs from nude mice in the orthotopic implantation assay (black arrows: metastatic nodules). **(C)** Box plot showing the numbers of lung nodules from the corresponding mice (mean ± SD, n = 8). **(D)** Representative images of H&E-stained histological sections of livers from nude mice in the orthotopic implantation assay (black arrows: metastatic nodules). **(E)** Box plot showing the numbers of liver metastatic nodules from the corresponding mice (mean ± SD, n = 8). Pseudocolor heat-maps indicate the intensity of bioluminescence from low (purple) to high (red) (mean ± SD, n = 8). One-way ANOVA was used for single-factor sample comparisons. ***P < 0.001 compared with ezrin-WT.

